# Synthesis of Sitagliptin Intermediate by a Multi-Enzymatic Cascade System Using Lipase and Transaminase With Benzylamine as an Amino Donor

**DOI:** 10.3389/fbioe.2021.757062

**Published:** 2021-10-06

**Authors:** Taresh P. Khobragade, Sharad Sarak, Amol D. Pagar, Hyunwoo Jeon, Pritam Giri, Hyungdon Yun

**Affiliations:** Department of Systems Biotechnology, Konkuk University, Seoul, South Korea

**Keywords:** sitagliptin, β-amino acid, transaminase, esterase, benzylamine, promoter engineering

## Abstract

Herein, we report the development of a multi-enzyme cascade using transaminase (TA), esterase, aldehyde reductase (AHR), and formate dehydrogenase (FDH), using benzylamine as an amino donor to synthesize the industrially important compound sitagliptin intermediate. A panel of 16 TAs was screened using ethyl 3-oxo-4-(2,4,5-trifluorophenyl) butanoate as a substrate (**1**). Amongst these enzymes, TA from *Roseomonas deserti* (TARO) was found to be the most suitable, showing the highest activity towards benzylamine (∼70%). The inhibitory effect of benzaldehyde was resolved by using AHR from *Synechocystis* sp. and FDH from *Pseudomonas* sp., which catalyzed the conversion of benzaldehyde to benzyl alcohol at the expense of NAD(P)H. Reaction parameters, such as pH, buffer system, and concentration of amino donor, were optimized. A single whole-cell system was developed for co-expressing TARO and esterase, and the promoter engineering strategy was adopted to control the expression level of each biocatalyst. The whole-cell reactions were performed with varying substrate concentrations (10–100 mM), resulting in excellent conversions (ranging from 72 to 91%) into the desired product. Finally, the applicability of this cascade was highlighted on Gram scale, indicating production of 70% of the sitagliptin intermediate with 61% isolated yield. The protocol reported herein may be considered an alternative to existing methods with respect to the use of cheaper amine donors as well as improved synthesis of (*R*) and (*S*) enantiomers with the use of non-chiral amino donors.

## Introduction

Biocatalysts continue to be explored for consideration as green alternatives to alleviate the environmental issues encountered with the use of methods pertaining to traditional organic chemistry (Patil et al., 2018; Pagar et al., 2021). Thus, multi-enzyme cascade reactions have garnered attention owing to their elegance (Sperl and Sieber, 2018; Hwang and Lee, 2019; Martínez-Montero et al., 2019), comprehensiveness, and involvement in the synthesis of a considerable variety of pharmaceuticals, agrochemicals, and other industrially important chemicals (Sung et al., 2018; Patil et al., 2019; Yoon et al., 2019).

Sitagliptin is a well-reported example of a drug containing a β-amino acid moiety as a key component. Sitagliptin, an inhibitor of dipeptidyl peptidase-4, is used as an oral antidiabetic drug to treat type 2 diabetes (Yun et al., 2004; Aschner et al., 2006; Desai, 2011; Villegas-Torres et al., 2015). Merck has described the industrial manufacturing method of sitagliptin phosphate. In the first-generation process (chemistry route), the chirality of achiral β-keto ester was introduced in the form of a hydroxy group in β-hydroxy acid through ruthenium-catalyzed asymmetric hydrogenation. The hydroxyl group was then transformed into protected amino acid through several steps, followed by direct coupling to triazolopiperazine to afford sitagliptin. The complete process involves eight steps to enable the production of sitagliptin phosphate with an overall yield of 52% (Hansen et al., 2005; Hansen et al., 2009). In comparison, the second-generation process comprised a three-step one-pot synthesis of dehydro-sitagliptin. The synthesis was initiated with trifluoro phenylacetic acid, followed by serial and controlled addition of other reactants and reagents, resulting in the formation of sitagliptin phosphate. In the last step, the product was crystallized and separated via simple filtration and resulted in the obtainment of 82% overall yield (Hsiao et al., 2004; Xu et al., 2004; Clausen et al., 2006).

The abovementioned chemical process involves the use of hazardous chemicals and other toxic substances. Biocatalysts are environmentally friendly and can be used as an alternative for the chemical process. TA is reportedly a useful and versatile biocatalyst for the selective and sustainable synthesis of amines, including ketones and aldehydes (Mathew and Yun, 2012; Park and Shin, 2013; Guo and Berglund, 2017; Slabu et al., 2017; Hauer, 2020). For the enzymatic synthesis of sitagliptin, the *R*-selective TA derived from *Arthrobacter* sp. was recently engineered using computational modeling and iterative directed evolution tools. The evolved enzyme harbors 27 mutations, highlighting it as a highly active variant, thus enabling its use in the production of the desired sitagliptin at 92% yield (Savile et al., 2010). Another enzymatic route considered for the synthesis of a sitagliptin intermediate using TA (ATA117-rd11 from *Arthrobacter* sp.) was reportedly adopted to produce the desired sitagliptin intermediate at a yield of 82% (Hou et al., 2016). Recently, Zheng and co-workers have reported the asymmetric synthesis of sitagliptin intermediate using TA engineered from *Arthrobacter cumminsii* ZJUT212 (M1+M122H), which helped obtain the target product at ∼92% yield (Cheng et al., 2020). Recently, we reported two enzymatic strategies for the synthesis of sitagliptin intermediate using TA from *Ilumatobacter coccineus* (TAIC) and commercial *Candida rugosa* lipase (CRL) in one cascade, as well as using TAIC and esterase from *Pseudomonas stutzeri* (Est PS) in another cascade. In both cascades, (*S*)-α-methylbenzylamine ((*S*)-α-MBA) was used as an amine donor, resulting in ∼82% conversion to the desired product (Kim et al., 2019; Khobragade et al., 2021).

Although these TAs are routinely used for the production of various industrially important compounds, including sitagliptin, they present with various inherent problems for large-scale production, such as inhibition of the biocatalyst by co-products and unfavorable thermodynamic equilibrium. (Koszelewski et al., 2010; Park et al., 2013; Voges et al., 2016). Therefore, selection of a suitable amine donor is a crucial step for efficient TA reactions. As such, L-alanine (Ala) has been considered as the most widely utilized amine donor ever since its deaminated product (i.e., pyruvate) was identified with the ability of being recycled. Several enzymes that demonstrate the ability to degrade pyruvate or to convert pyruvate into Ala have been studied, including lactate dehydrogenase, pyruvate decarboxylase, acetolactate synthase, and alanine dehydrogenase, complemented by cofactor recycling using FDH or glucose dehydrogenase (Shin and Kim, 2001; Hoehne et al., 2008; Koszelewski et al., 2008; Koszelewski et al., 2008; Truppo et al., 2009; Truppo et al., 2009; Koszelewski et al., 2010; Mutti et al., 2011; Schätzle et al., 2011; Mutti et al., 2012; Pressnitz et al., 2013; Sayer et al., 2014). Moreover, to shift the reaction equilibrium, a higher amount of Ala was used, and additional enzymes (auxiliary enzymes) were introduced to remove by-products and to recycle the cofactor. However, owing to the high overall cost, this approach is not appealing for industrial applications. Isopropyl amine (IPA) is considered a promising amino donor, as many TAs demonstrate the ability to accept IPA as an efficient amino donor, including *A. citreus* TA mutant, ATA117 mutant, and TAs from *P. aeruginosa, P. denitrificans,* and *A. terreus* (Cassimjee et al., 2010; Savile et al., 2010; Fesko et al., 2013; Park et al., 2013; Simon et al., 2014; Dawood et al., 2018). The advantages of IPA include its achiral nature, which enable its application with either (*S*)- or (*R*)-selective TAs, and the easy elimination of acetone via implementation of physical or chemical approaches (Kelefiotis-Stratidakis et al., 2019). However, IPA also presents with a few limitations, such as the disruption of reactions for final product isolation and purification in the presence of excess amounts of IPA, and the additional assembly required to remove the deaminated product. Recently, ‘smart’ amine donors such as 3-amino-cyclohexa-1,5-dienecarboxylic acid, o-xylylene diamine, cis-but-2-ene-1,4-diol, cis-but-2-ene-1,4-diamine, and cadaverine have been reported as effective amine donors because they can be used at equimolar quantities (Wang et al., 2013; Green et al., 2014; Gomm et al., 2016; Martínez-Montero et al., 2016; Payer et al., 2017). One of the advantages of these amino donors is the auto-degrading properties of their co-products. However, the separation of product and substrate upon reaction completion can be challenging owing to the similar molecular properties of the amino donors. Moreover, they are costly compared to Ala and IPA. Currently, (*S*)-α-MBA is the most commonly used amine donor for the synthesis of β-amino acids, and the majority of TAs display high activity towards (*S*)-α-MBA, but (*S*)-α-MBA is a relatively expensive co-substrate for transamination reaction; furthermore, the deaminated co-product, acetophenone, is known to inhibit TA (Shon et al., 2014; Mathew et al., 2016). Nevertheless, in a study investigating the amino donor specificity of TA derived from *Vibrio fluvialis* JS17, the biocatalyst exhibited 3.6 and 30% specificity for IPA and Ala, respectively, compared with (*S*)-α-MBA (100%), and benzylamine showed 1.15-fold higher activity than (*S*)-α-MBA (Shin and Kim, 2002)).

**FIGURE 1:**
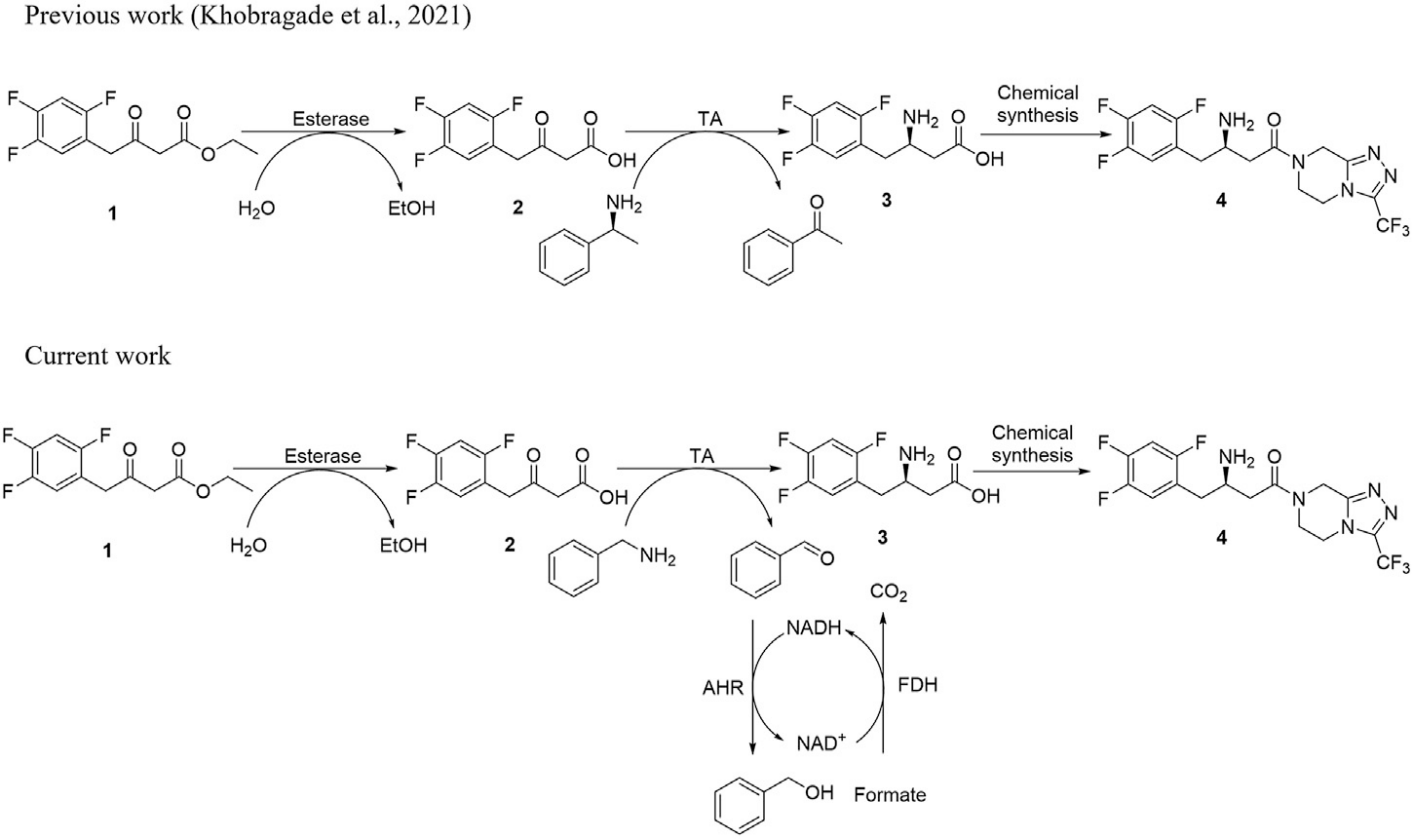
**(A)** Schematic diagram for the synthesis of sitagliptin phosphate from β-keto ester substrate using (*S*)-α-MBA as an amino donor. **(B)** Schematic diagram for the synthesis of sitagliptin intermediate using benzylamine as an amine donor with the AHR/FDH system.

However, studies conducted for screening and designing efficient amine donors for the synthesis of β-amino acids compared to chiral amines remains limited. Therefore, exploration of a suitable and cheaper amine donor to shift the reaction equilibrium towards product formation has emerged as a key step in the establishment of multi-enzyme cascade reactions. Benzylamine is considered the cheapest donor among the abovementioned amino donors. Furthermore, it can be used to perform screening of both (*S*)- and (*R*)-selective TAs. Therefore, as a continuation to our previously published work (Kim et al., 2019; Khobragade et al., 2021), the present study was conducted to investigate the effectiveness of benzylamine as an amino donor for the synthesis of sitagliptin intermediate by using **1** as the substrate (Figure 1). First, we selected a panel of 16 in-house TAs and assessed their specificity for benzylamine. To shift the reaction equilibrium towards product formation and to minimize enzyme inhibition by the action of the deaminated co-products, the AHR/FDH system was used. In this system, AHR has been considered to catalyze the conversion of benzaldehyde into benzyl alcohol at the expense of NAD(P)H cofactor, and FDH has been considered to supply the depleted NAD(P)H by recycling the corresponding NAD(P)+ cofactor.

## Materials and Methods

### Chemicals and Enzymes

*Candida rugosa* lipase (Catalog No. L1754), Benzylamine (*S*)-α-methylbenzylamine [(*S*)-α-MBA], 2,3,4,6-Tetra-O-acetyl-β-D-glucopyranosyl isothiocyanate (IPTG), pyridoxal 5′-phosphate hydrate (PLP), perchloric acid (PCA), dimethyl sulfoxide (DMSO) were purchased from Sigma-Aldrich (Sigma-Aldrich Korea, Seoul, South Korea). Ethyl 3-oxo-4-(2,4,5-trifluorophenyl) butanoate was obtained from CKD BiO Corp, Korea.

### Expression of the Enzymes

The genes encoding various TAs, AHR, and FDH were synthesized by Bionics (Seoul, Korea). Then gene was inserted into IPTG-inducible pET 24ma vectors (TAs). The AHR and FDH were cloned pET duet vectors (AHR in MCS I site whereas FDH in MCS II site). The plasmids were transformed to *Escherichia coli* (BL21) cells, and the transformants were grown in 0.5 L LB-containing kanamycin or ampicillin (50 or 100 μg/ml) at 37°C. The cells were induced with 0.1 mM IPTG when OD_600_ reached 0.6–0.8 (Kim et al., 2019; Shon et al., 2014; Mathew et al., 2016.). After the overnight induction at 20°C, the cells were harvested by centrifugation (5,000 rpm, 10 min) and washed twice with 200 mM Tris/HCl buffer (pH 8.0).

### Determination of Specific Activity of Est PS Using Whole Cells

To examine the specific activity of Est PS, cells were grown in 0.2 L of LB media containing ampicillin (100 μg/ml) at 37°C. When the cell OD_600_ reached 0.6–0.8, 0.1 mM IPTG was added (Kim et al., 2019.), followed by overnight incubation at 20°C. Thereafter, the cells were harvested by centrifugation (4,400 × *g*, 10 min) and were subjected to washing steps two times using 200 mM Tris/HCl buffer (pH 8.0). For the reaction, Est PS (0.15 mg_DCW_/ml) was incubated at 37°C for 20 min with DMSO 15% and 500 µl Tris-HCl buffer (200 mM, pH 7.0) containing ethyl 3-oxo-4-(2,4,5-trifluorophenyl) butanoate (10 mM) as a substrate. The reaction was terminated by adding 1% perchloric acid at a ratio of 1:1 (v/v). Next, the reaction mixture was centrifuged at 17,000 × *g* for 30 min, and the clear supernatant was analyzed via high-performance liquid chromatography (HPLC) using a C 18 symmetry column.

### Whole-Cell Biotransformation Reactions of the Substrate at Different Concentrations

Whole-cell biotransformation reactions were conducted in a reaction volume (5 ml) containing **1** (10 or 50 mM), benzylamine (20 or 150 mM), DMSO (5% (v/v), PLP (0.5 mM), CRL (10 or 50 mg/ml), Tris-HCl buffer (200 mM, pH 8.0), and whole cells expressing TA only or co-expressing TARO-Est PS. During the reaction, pH was reduced owing to the hydrolysis of an ester substrate. Therefore, the pH of the reaction mixture was adjusted manually by adding 5 M NaOH solution at specific intervals during the 5-h reaction. Subsequently, the sample was collected, and the presence of **3** was detected using HPLC.

### Construction of Different Variant of T7 Promoter

The promotor engineering strategy is applied to achieve the optimum expression level of Est PS as described in our previous study (Khobragade et al., 2021). In brief, the gene (Est PS) was cloned in the different strengths of T7 promotors in the pET15b plasmid (Chizzolini et al., 2014). The newly constructed different variant of pET15b plasmid and TARO was co-expressed to develop a single-cell biocatalyst. The specific activity and enzymatic reaction of newly developed co-expressed whole cell was mentioned above.

### Whole-Cell Biotransformation Reaction of the Substrate at Different Concentrations Using a pH-Stat System

Whole-cell biotransformation reactions were performed in a reaction volume (30 ml) comprising **1** (100–400 mM), benzylamine (150–300 mM), DMSO (5% (v/v), PLP (0.5 mM), Tris-HCl buffer (200 mM, pH 8.0), and cells co-expressing TARO-Est PS. Similar to the above-mentioned reaction, the hydrolysis of an ester substrate to its corresponding acid caused a decrease in pH in this reaction. Therefore, an 877 Titrino Plus pH-stat system (Metrohm AG, Herisau, Switzerland) for automatic titration with 5 M NaOH was used to ensure a stable pH. Samples were collected at specific intervals for the identification of **3** using HPLC.

### Analytical Method

The quantitative analysis of β-amino acid was performed using HPLC with a Crownpak CR column (Daicel Co., Japan) at 210 nm, with an elution of perchloric acid solution (pH 1.5; 0.6 ml/min) as previously reported (Kim et al., 2019; Shon et al., 2014). The β-keto ester (**1**) were analyzed using HPLC with C-18 Column. Mobile phase: 40% water and 60% methanol containing 0.1 TFA. Flowrate: 1.0 ml/min, wavelength: 254 nm.

## Results and Discussion

### Screening of TAs for the Synthesis of Sitagliptin Intermediate from β-keto Acid

**FIGURE 2:**
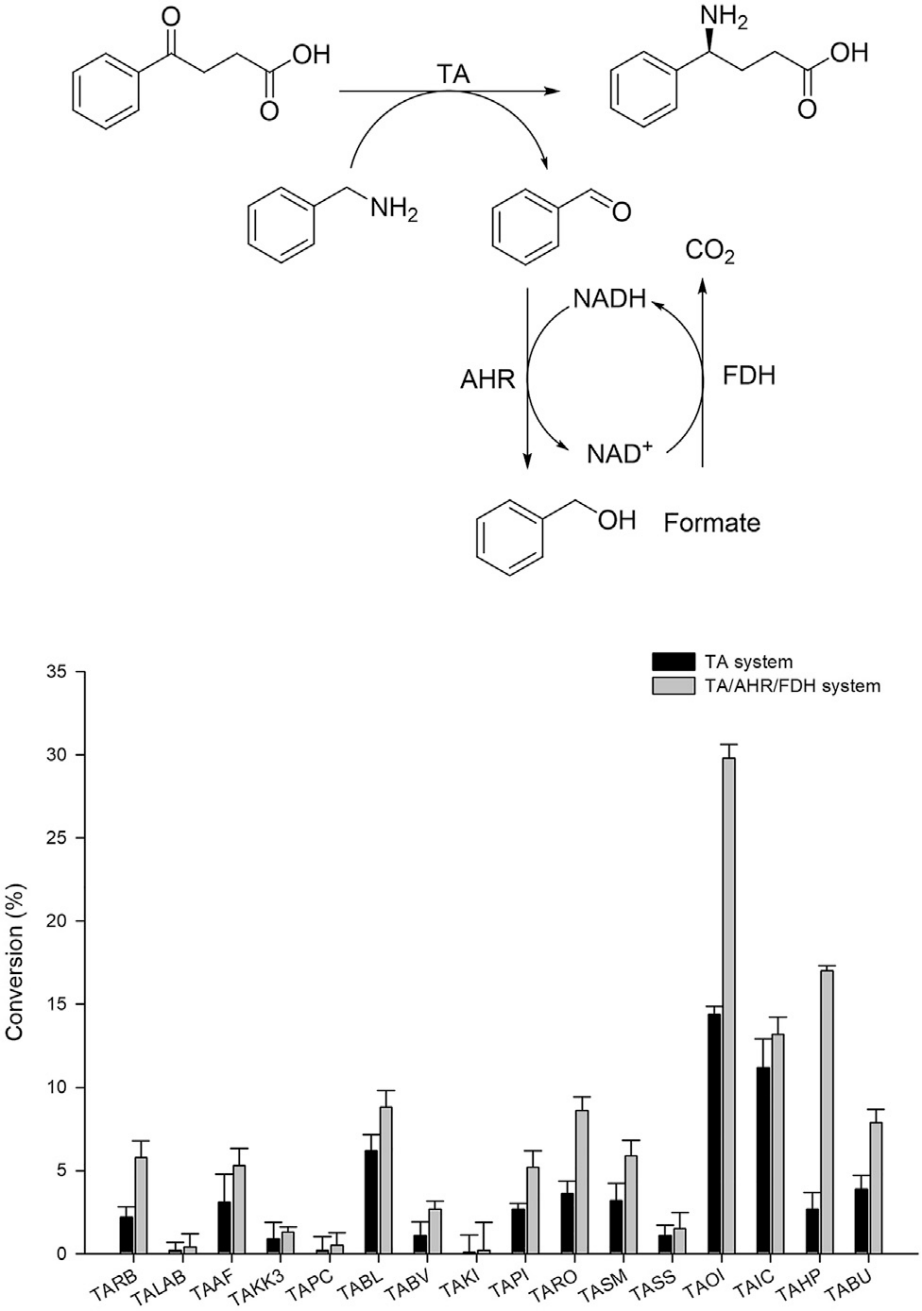
Screening of TAs using benzylamine as an amino donor with TA and the TA/AHR/FDH system. Reaction conditions: 10 mM 4-oxo-4-phenylbutanoic acid, 20 mM benzylamine, 0.1 mM PLP, 9 mg_CDW_/ml TAs or 9 mg_CDW_/ml AHR/FDH cells.

Generally, (*S*)-selective TAs with specificity for Ala as an amine donor belong to fold-type l (the aspartate aminotransferase superfamily). Among these enzymes (*S*)-β-TAs reportedly possess high activity towards β- and γ-keto acids as a substrate, resulting in the corresponding β- and γ-amino acids. These amine compounds are extensively used for the synthesis of many industrially relevant chiral compounds. Earlier studies have proven that TA exhibits good reactivity towards (*S*)-α-MBA as an amine donor for the transamination step (Mathew et al., 2016; Kim et al., 2019). For screening of suitable TA biocatalysts with high specificity for benzylamine, we selected a panel of our in-house TAs as described previously (Kim et al., 2019). According to previous studies, TAs demonstrating specificity for β-keto acids also possess good reactivity to γ-keto acids, enabling the establishment of improved assay conditions for analysis of γ-amino acids. Therefore, for the initial screening of TAs, 4-oxo-4-phenylbutanoic acid was selected as a substrate, and benzylamine was used as an amine donor. For convenience, whole-cell biocatalysts were used in this assay. Briefly, codon-optimized genes encoding the suitable TAs from 16 desired sources (Table 1) were synthesized and cloned into pET24ma vectors and were then expressed using *E. coli* BL21 (DE3) host cells (Table 1). Whole-cell biotransformation was performed using 10 mM 4-oxo-4-phenylbutanoic acid substrate, 20 mM benzylamine, 0.5 mM PLP, and 9 mg_CDW_/ml cells of each TA biocatalyst (Figure 2). Among the sixteen enzymes investigated, TAOI and TAIC exhibited the highest conversion 13–15%, whereas eight other TA biocatalysts (TABL, TABU, TARO, TASM, TAHP, TARB, TAAF, and TAPI) led to the achievement of modest conversion of 2–7% into the desired product. On the contrary, the remaining six TAs (TALAB, TAKK3, TAPC, TABV, TAKI, and TASS) resulted in the obtainment of a negligible amount of the desired product (<0.5%). Although few TAs showed comparable activity, the considerably lower conversion could be attributable to enzyme inhibition by benzaldehyde, which was generated as a deaminated co-product of benzylamine during the reaction. However, we examined the enzyme inhibition of benzaldehyde with highly active TAs like TAIC, TARO, and TAOI. As expected, we observed the inhibition of benzaldehyde on the enzyme (Supplementary Figure S1). Therefore, we established an aldehyde removal system to minimize the inhibition of TA biocatalyst and to shift the reaction equilibrium towards product formation. Literature reports have evidenced that AHR demonstrates good activity towards benzaldehyde and can efficiently catalyze its conversion into benzyl alcohol at the expense of NAD(P)H; thus, we selected AHR and FDH for recycling of the depleted NAD(P)H cofactor. With the adoption of such a removal system, the reactions were performed using 10 mM 4-oxo-4-phenylbutanoic acid, 20 mM benzylamine, 0.5 mM PLP, and 9 mg_CDW_/ml of each TA biocatalyst and AHR/FDH cells (Figure 2). As expected, utilization of the benzaldehyde removal system improved product formation under conditions of TAOI (∼30%) catalysis by 2-fold. Interestingly, product formation via catalysis by TARO and TAHP was significantly enhanced by ∼3- and 6-fold, respectively. In contrast, all other TAs did not significantly elevate product formation, resulting in the achievement of only <14% yield of the desired product. These results suggested that benzaldehyde exerted inhibitory effects against the TAs and should be removed to efficiently improve TA performance. The AHR/FDH system adopted for benzaldehyde removal successfully improved the overall reaction conversion and was thus used for further optimization of the cascade reaction. Finally, the best six TAs (TAHP, TARO, TAOI, TAIC, TABL, and TABV) from the panel were selected for subsequent optimization using coupled enzyme reaction.

**TABLE 1:** Various enzymes used in this study.

Entry	Enzyme	Assigned name	GenBank identification number	References
1	*Rhodospirillaceae bacterium*	TARB	WP 092830126.1	Kim et al. (2019)
2	*Labrenziasp-*LAB	TALAB	WP 0841772225.1	
3	*Afipia* sp. P52-10	TAAF	WP 051444297.1	
4	*Oceanibaculum indicum*	TAOI	WP 008945048.1	
5	*Ilumatobacter coccineus*	TAIC	WP 015443725.1	
6	*Variovorax* sp. KK3	TAKK3	WP 076998155.1	
7	*Paraburkholderia caribensis*	TAPC	AMV 45988.1	
8	*Hydrogenophaga palleronii*	TAHP	WP 066271212.1	
9	*Solirubrobacter soli*	TASS	WP 028067622.1	
10	*Kineosporia* sp. *R_H_3*	TAKI	WP 088319003.1	
11	*Roseomonas deserti*	TARO	WP 076960246.1	
12	*Sinorhizobium meliloti*	TASM	WP 018099655.1	
13	*Bosea lupine*	TABL	WP 091836827.1	
14	*Bosea vaviloviae*	TABV	WP 069688334.1	
15	*Pseudacidovorax intermedius*	TAPI	WP 052383549.1	
16	*Burkholderia* sp. *UYPR1.413*	TABU	WP 028368728.1	
17	*Synechocystis* sp	AHR	WP_028949165.1	
18	*Pseudomonas* sp. *101*	FDH	P33160.3	
19	*Pseudomonas stutzeri*	Est PS	WP_020308675.1	Khobragade et al. (2021)

### Design and Optimization of Coupled Enzyme Reaction (Transaminase/Lipase) for the Synthesis of Sitagliptin Intermediate From the Corresponding β-keto Ester

**FIGURE 3:**
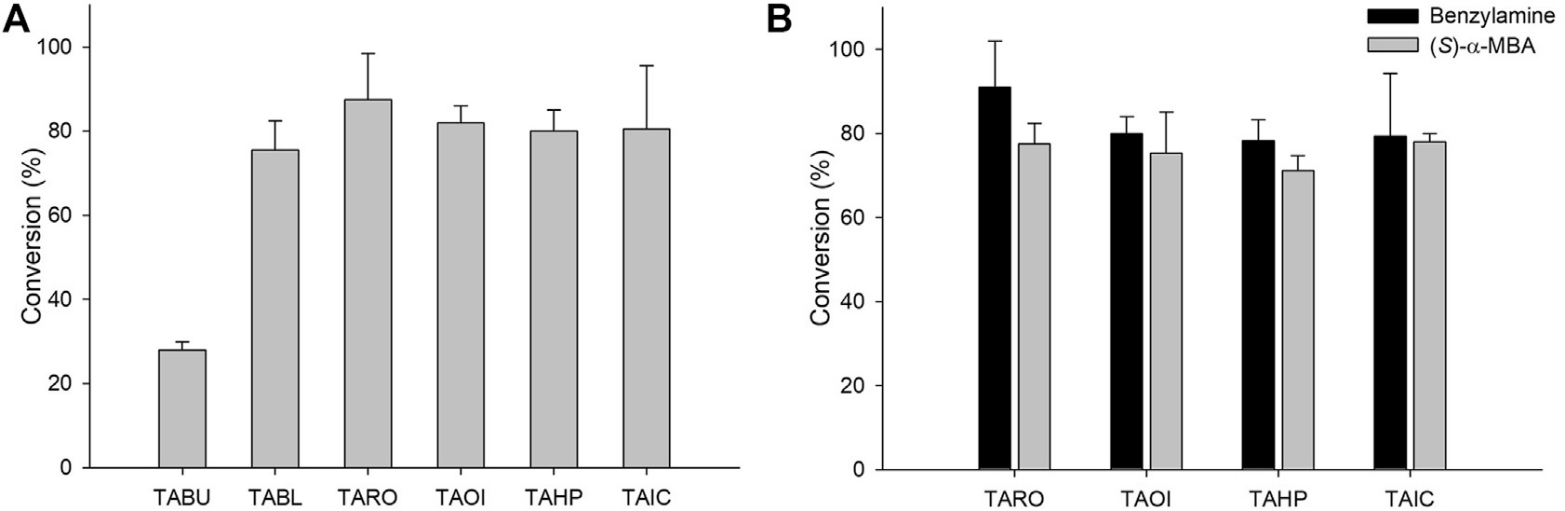
**(A)** Synthesis of sitagliptin intermediate using the TA/CRL/AHR/FDH system. Reaction conditions: 10 mM **1**, 20 mM benzylamine, 10 mg/ml CRL, 9 mg_CDW_/ml TAs, 9 mg_CDW_/ml AHR/FDH cells. **(B)** Comparison of benzylamine and (*S*)-α-MBA as amino donors. Reaction conditions for using benzylamine as an amine donor: 10 mM 1, 20 mM benzylamine, 0.5 mM PLP, 10 mg/ml CRL, 9 mg_CDW_/ml TAs, 9 mg_CDW_/ml AHR/FDH, 50 mM sodium formate, 200 mM Tris-HCl Buffer (pH 8.0), 37°C. Reaction conditions for using (*S*)-α-MBA as an amine donor: 10 mM 1, 20 mM (*S*)-α-MBA, 0.5 mM PLP, 10 mg/ml CRL, 9 mg_CDW_/ml TAs, 200 mM Tris-HCl Buffer (pH 8.0), 37°C.

For the synthesis of sitagliptin intermediate using coupled enzyme reaction, lipase is necessary for hydrolysis of the ester substrate. Previous studies have reported the efficacy of CRL for hydrolysis of an ester substrate. Additionally, to overcome the low intrinsic stability of β-keto acids due to rapid decarboxylation (Rudat et al., 2012), many biocatalytic approaches have been developed by designing the corresponding ester or nitriles as a substrate. Notably, rapid hydrolysis of an ester substrate will cause pH decrease during the reaction, which will affect the optimal conditions of the biocatalysts in the cascade. Therefore, the decreased pH value should be compensated by adding a suitable concentration of base (5 M NaOH) at various time intervals. Considering this, coupled reactions consisting of CRL and six TAs were performed using 10 mM **1** as a substrate. In these reactions, TARO was found to be the best TA, presenting with a conversion of 91%. The other three enzymes exhibited the achievement of 75–82% conversion into the desired product (Figure 3A **)**. These results suggested that all selected enzymes except TABU demonstrated a higher specificity for β-keto acid than that for γ-keto acid, and they were therefore selected for further optimization. Next, the reaction parameters, such as pH and buffer system, were optimized for reaction with the four selected TAs (TARO, TAHP, TAOI, and TAIC) (Supplementary Figure S2). To determine the optimum pH and suitable buffer species for reaction, three different buffer systems were inspected, namely acetate (pH 4.0–6.0), phosphate (pH 6.0–8.0), and Tris-HCl (pH 7.0–10.0). All four TAs showed the highest activity at pH 8.0 (Tris-HCl buffer); however, for TA from *Vibrio fluvialis* JS17, slightly alkaline pH conditions were necessary, possibly owing to an increase in the effective concentration of deprotonated amine donor, which enhanced the external aldimine formation (Guo and Berglund, 2017).

After optimization of the reaction, we compared the specificity of these TAs for (*S*)-α-MBA. For reactions using benzylamine as an amine donor, the AHR/FDH system was used to remove the benzaldehyde. However, the system was not adopted in reactions with (*S*)-α-MBA used as an amine donor because the removal of acetophenone did not reportedly improve product formation (Khobragade et al., 2021). The reactions consisted of 10 mM **1**, 20 mM benzylamine, 10 mg/ml CRL, 0.5 mM PLP, and 9 mg_CDW_/ml of each biocatalyst. In reactions using benzylamine as an amine donor, all four TAs led to the obtainment of >80% yield, with TARO showing the highest conversion of 90%. In contrast, reactions using (*S*)-α-MBA led to the achievement of the target product at only ∼70% yield, with TARO exhibiting the highest conversion of 77% (Figure 3B). The observed 10–15% higher conversion with benzylamine as an amine donor indicated that the four selected TAs possessed higher specificity for benzylamine than that for (*S*)-α-MBA. Encouraged by these results, we performed further reactions with increased substrate concentration (50 mM **1**) and varying CRL concentrations (30–60 mg/ml). Among the four enzymes, TAHP demonstrated the highest conversion of 36% with 40 mg/ml CRL, whereas the other three enzymes resulted in the achievement of <26% yield, with TARO displaying the second-highest conversion (26% at 40 mg/ml of CRL). Therefore, TAOI and TAIC were eliminated, and TAHP and TARO were selected for subsequent studies.

In general, the amount of amine donor plays a vital role in transamination reaction. Increasing the amount of amine donor in the reaction is a commonly used strategy for shifting reaction equilibrium towards product formation. However, such a high amount of amine donor can adversely affect the activity of other enzyme biocatalysts or TA itself in the cascade (Kim et al., 2019). Hence, to determine the optimal concentration of amine donor, reactions were conducted using varying concentrations of benzylamine with 50 mM **1** and 27 mg_CDW_/mL biocatalyst (Table 2). Benzylamine at a concentration of 150 mM (3-fold increase in concentration) was found to be optimal for the reaction, resulting in 38 and 34% conversion into the desired product (**3**) by TARO and TAHP, respectively (Table 2, entry 3). On the contrary, further increases in the amount of benzylamine (200–300 mM) led to lower product formation (<30%), indicating that a concentration of more than 150 mM benzylamine inhibited the activity of the enzyme biocatalyst in the cascade. Furthermore, to confirm benzylamine consumption during the reaction, we determined the amount of benzyl alcohol produced. The results suggested that equal amount of the product was formed and amine donor was consumed in the reaction (Supplementary Figure S3). Our finding also indicated that the AHR/FDH system could be used to successfully convert the benzaldehyde into benzyl alcohol, thereby shifting the reaction equilibrium towards product formation. Additionally, we examined the effect of different amino donor systems (benzylamine, D-alanine, and IPA) on an (*R*)-selective TA from *Neosartorya fischeri*. The benzylamine amino donor system provided the highest conversion (18%), whereas the D-alanine and IPA amino donor systems resulted in negligible conversion (<0.1%) (Supplementary Table S1). Therefore, the benzylamine donor system presented with applicability for both of (*R*)- and (*S*)-selective TAs.

**TABLE 2:** Optimization of benzylamine concentration using the TA/CRL/AHR/FDH system.

Entry	Substrate (mM)	Benzylamine (mM)	TARO or TAHP (mg_CDW_/ml)	Substrate: Donor ratio	Conv. (%)^a^ TARO or TAHP	Reaction time (h)
1	50	50	27	1:1	15 ± 4 or 12 ± 2	24
2	50	100	27	1:2	23 ± 2 or 26 ± 7	24
3	50	150	27	1:3	36 ± 5 or 33 ± 9	24
4	50	200	27	1:4	29 ± 4 or 28 ± 4	24
5	50	250	27	1:5	23 ± 2 or 25 ± 5	24
6	50	300	27	1:6	18 ± 3 or 14 ± 8	24

Reaction performed containing 200 mM Tris-HCl buffer (pH 8.0), 0.5 mM PLP, 40 mg/ml CRL, 27 mg_CDW_/ml AHR/FDH, 100 mM sodium formate; temperature: 37°C.

a HPLC analysis.

### Application of Promoter Engineering Strategy for the Synthesis of Sitagliptin Intermediate

Single whole-cell biocatalysts co-expressing multiple enzymes are advantageous in terms of convenient handling and enhanced mass transfer efficiency (Tufvesson et al., 2014; Iglesias et al., 2017; Telzerow et al., 2019). In a previous study, we reported that ratio of esterase to TA should be balanced for fine-tuning of the activity (TAIC-Est PS). Therefore, promotor engineering strategy was developed and applied to control the expression levels of each enzyme (Chizzolini et al., 2014; Yoo et al., 2019). The Est PS used in the study had a specific activity of 3.96 U/mg, which was >12-fold higher than that of CRL. In this experiment, we used a similar strategy by replacing CRL with a biocatalyst co-expressing TARO and Est PS.

**FIGURE 4:**
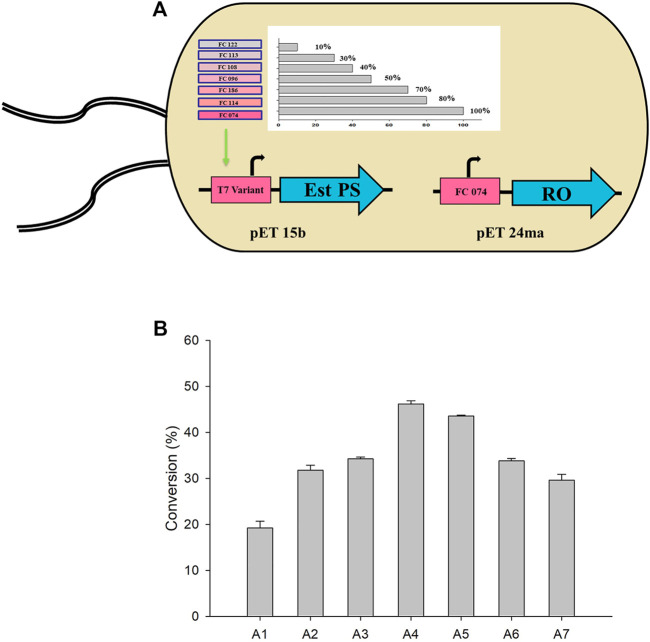
**(A)** Single-cell system co-expressing different variants of Est PS and TARO. **(B)** Whole-cell biotransformation with various engineered systems for the synthesis of sitagliptin intermediate. Reaction conditions: 50 mM **1**, 150 mM benzylamine, 0.5 mM PLP, 27 mg_CDW_/ml TARO-Est PS, 27 mg_CDW_/ml AHR/FDH, 100 mM sodium formate, 200 mM Tris-HCl Buffer (pH 8.0); temperature: 37°C.

Therefore, genes encoding TARO and Est PS were designed and cloned to pET24ma and pET15b vectors, respectively. Whole-cell *E. coli* strains co-expressing variants of Est PS with different promoter strengths and TARO were also developed (Figure 4A). The specific activity of the cells expressing TARO and Est PS alone was estimated to be 0.014 U/mg and 3.56 U/mg, respectively. Similarly, the specific activity of whole cells co-expressing TARO-Est PS was measured (Table 3), and the results showed that the specific activity decreased with decreasing order of Est PS promotor strength, indicating reduced expression level of the enzyme; in contrast, the specific activity of TARO was enhanced, suggesting that TARO expression was higher than that of Est PS in the single-cell system. For Est PS, the highest specific activity (1.69 U/mg) was achieved with the A1 system, and the lowest activity (0.44 U/mg) was obtained with the A7 system. On the contrary, for TARO, the A7 system exhibited the highest activity of 0.034 U/mg and A1 demonstrated the lowest activity of 0.017 U/mg (Table 3 **,** entry 1–7).

**TABLE 3:** The co-expression system of TARO and Est PS with different T7 promoter variants.

Entry	Engineered systems	Promoter		Specific activity (U/mg)	
		T7 promoter variant	Relative strength (%)^a^	TARO^b^	Est PS^c^
1	A1	FC 074	100	0.017 ± 0.003	1.69 ± 0.06
2	A2	FC 114	80	0.021 ± 0.002	1.33 ± 0.03
3	A3	FC 186	70	0.023 ± 0.003	1.17 ± 0.04
4	A4	FC 096	50	0.026 ± 0.003	0.97 ± 0.04
5	A5	FC 108	40	0.029 ± 0.002	0.87 ± 0.07
6	A6	FC 113	30	0.031 ± 0.002	0.56 ± 0.03
7	A7	FC 122	10	0.034 ± 0.003	0.44 ± 0.06

a Strength of promoter (Chizzolini et al., 2014).

b Activity was measured using 200 mM Tris-HCl buffer (pH 7.0) containing 10 mM pyruvate and 20 mM benzylamine at 37°C.

c Activity was measured using 200 mM Tris-HCl buffer (pH 7.0) containing 10 mM 1 and 15% DMSO at 37°C.

Considering the findings obtained herein, subsequent whole-cell reactions were performed to determine the optimum single-cell system for the synthesis of sitagliptin intermediate. The reactions were conducted using 50 mM of **1**, 150 mM of benzylamine, and 27 mg_CDW_/ml of each cell system (A1-A7) (Figure 4B). The highest yield of 46% of the desired product **3**) was obtained by using the A4 system, whereas use of the A2, A3, A5, and A7 systems led to the achievement of moderate conversion of ∼27–34%. Conversely, the A1 system exhibited extremely low product formation (11%). Therefore, the A4 system was selected for further optimization.

**FIGURE 5:**
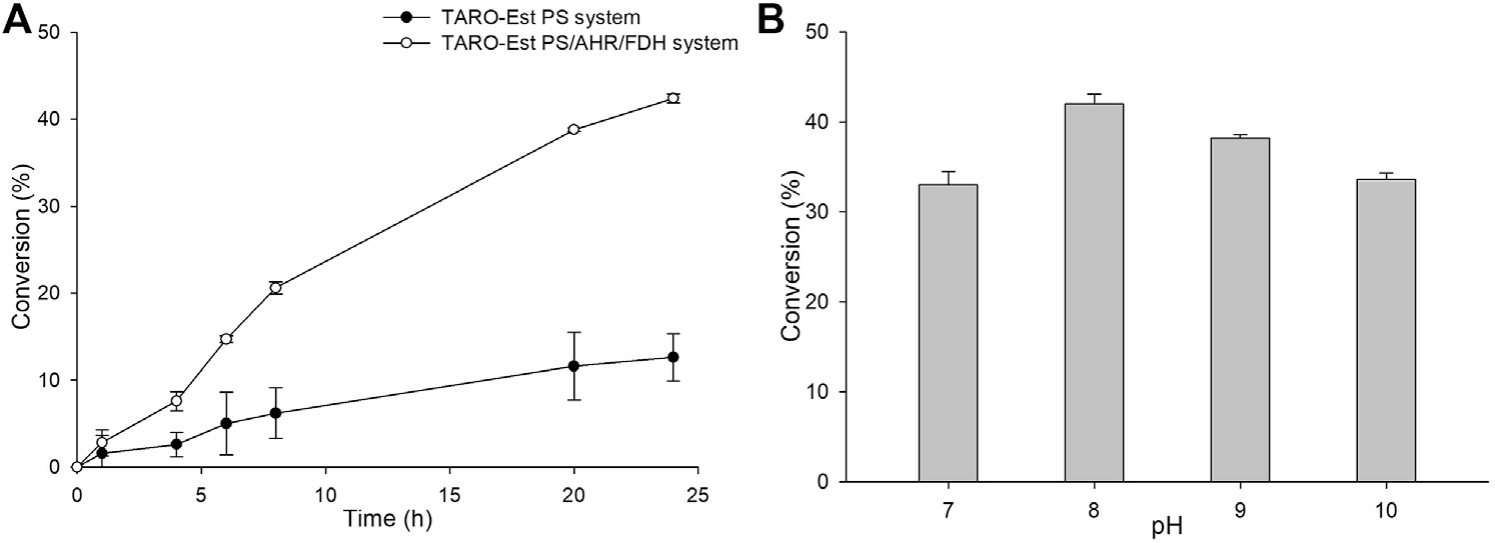
**(A)** Whole-cell biotransformation reaction with TARO-Est PS and the TARO-Est PS/AHR/FDH system for the synthesis of sitagliptin intermediate. Reaction conditions: 50 mM **1**, 150 mM benzylamine, 0.5 mM PLP, 27 mg_CDW_/ml TARO-Est PS or 27 mg_CDW_/ml AHR/FDH or 100 mM sodium formate, 200 mM Tris-HCl Buffer (pH 8.0); temperature: 37°C. **(B)** Whole-cell reaction for the synthesis of sitagliptin intermediate using different pH. Reaction conditions: 50 mM 1, 150 mM benzylamine, 0.5 mM PLP, 27 mg_CDW_/ml TARO-Est PS, 27 mg_CDW_/ml AHR/FDH, 100 mM sodium formate; temperature: 37°C.

Furthermore, to improve the reaction productivity, other reaction parameters, such as co-solvents and the loading amount of the whole-cell biocatalyst, were optimized (Supplementary Figure S4). Our results suggested that 5% (v/v) of DMSO and 27 mg_CDW_/mL of whole cells were optimum, resulting in the achievement of ∼44% yield of the sitagliptin intermediate. In comparison, the same reaction in the absence of AHR/FDH system led to the achievement of a significantly lower conversion rate of 10% into the desired product (**3**) (Figure 5A), further confirming that benzaldehyde inhibited the action of the enzyme biocatalyst. Next, as a key factor affecting the productivity of the reaction, pH was optimized by performing the reactions at a pH range of 7.0–10 with 200 mM of Tris-HCl buffer, 50 mM of **1,** and 27 mg_CDW_/ml of the biocatalyst. A pH value of 8.0 was found to be suitable, leading to the highest conversion rate of 42%, whereas alkaline pH values of 9.0 and 10 resulted in the achievement of only 38 and 34% conversion rates, respectively.

### Synthesis of Sitagliptin Intermediate Using the pH-Stat System

**FIGURE 6:**
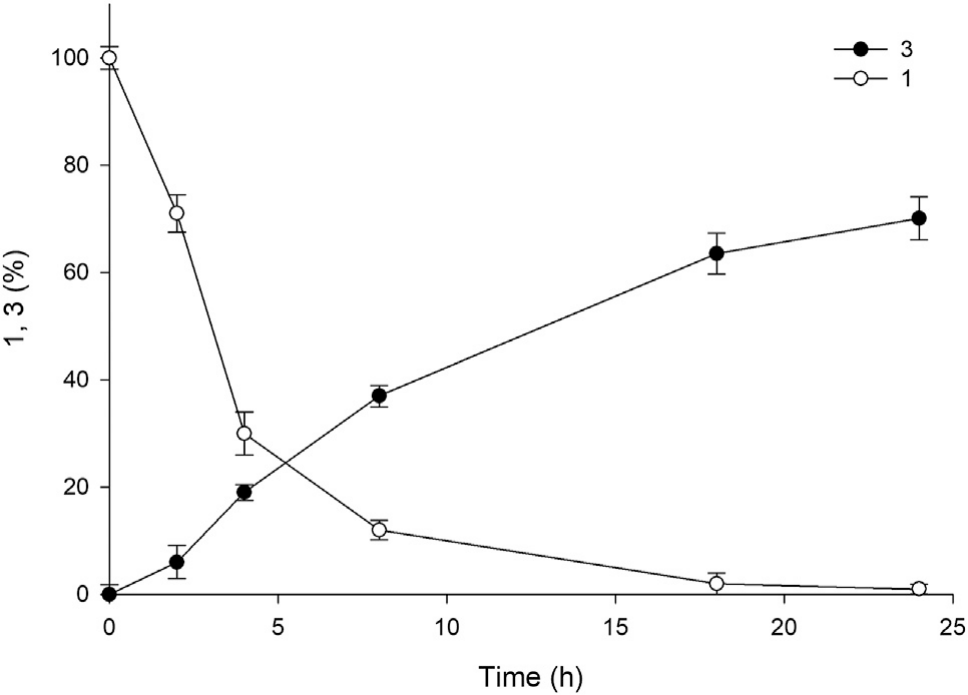
Time course of the reaction using 100 mM **1** for the synthesis of sitagliptin intermediate. Reaction conditions: 100 mM **1**, 300 mM benzylamine, 0.5 mM PLP, 60 mg_CDW_/mL TARO-Est PS, 60 mg_CDW_/mL AHR/FDH, 200 mM sodium formate, 200 mM Tris-HCl Buffer (pH 8.0); temperature: 37°C.

As mentioned above, pH plays a crucial role in the reaction, and the value consistently decreases owing the hydrolysis of ester into a carboxylic acid group. The pH values of the reactions in earlier experiments were controlled using 5 M NaOH via manual addition at an interval of 30 min for 5 h. However, the hydrolysis reaction catalyzed by esterase occurs more rapidly, resulting in a rapid decline in pH during the reaction. Therefore, manual pH adjustment is inefficient. Hence, to minimize pH fluctuation and to maintain the optimal pH value (8.0), we used a pH-stat system in the subsequent experiments. The reactions were performed using three systems (A1, A4, and A6) with 50 mM of **1** in 30 ml of the total reaction volume (Supplemenatry Figure S5). Sample analysis at various time points revealed almost complete consumption of the substrate within 18 h, resulting in the achievement of ∼80% yield of the desired product. The use of such a pH-stat system enhanced production by almost 50%, indicating that a fine-tuned control of pH is necessary for optimum product formation. The concentration of benzyl alcohol formed was comparable to the amount of product obtained (79%), indicating that an equal amount of amine donor was consumed during the reaction. On the contrary, A1 and A6 exhibited 36 and 56% yield of the sitagliptin intermediate, respectively, suggesting that the promoter engineering strategy enabled fine-tuned control of the expression level of each biocatalyst, which was beneficial for improving the reaction productivity (Supplemenatry Figure S6). Encouraged by these results, we selected the A4 system for conducting further reactions with elevated substrate concentrations of 100–200 mM of **1**. Our findings showed that the reaction with 100 mM substrate provided 72% yield of the sitagliptin intermediate and led to the achievement of nearly equal amount of benzyl alcohol (∼71%) (Figure 6), whereas reaction with 200 mM substrate rection produced only 37% yield of the desired product (Supplementary Figure S7).

Finally, the applicability of this coupled enzyme reaction was investigated on Gram scale via utilization of the A4 system using 100 mM (1.0 g) of substrate in 40 ml of the reaction volume. This preparative scale reaction led to the achievement of ∼70% yield of the sitagliptin intermediate (70 mM; 0.7 g) without major accumulation of reaction intermediates. After successful completion of the reaction, the sitagliptin intermediate was isolated and purified. First, the reaction mixture containing ∼0.7 g of the desired product was acidified using 5°N HCl (pH 2.0) to terminate the reaction, and the enzyme biocatalysts were precipitated. The reaction mixture was then centrifuged for 15 min to remove the cell mass, and the supernatant was subsequently neutralized by using 5 M NaOH and was then extracted with ethyl acetate (2 times) to remove the unreacted amine donor and other reaction intermediates that are soluble in organic solvents. Next, the separated aqueous layer was evaporated to dryness to obtain the crude product mixture, which was then solubilized into methanol to separate the buffer salts and other aqueous impurities. Finally, this methanol solution was concentrated to obtain the desired product, which was further purified by recrystallization using an ethanol:water system, yielding a pure white solid. The solid was then characterized by using ^1^H NMR and HPLC (61% isolated yield).

## Conclusion

In conclusion, we report benzylamine as an efficient alternative amine donor for transamination in the synthesis of sitagliptin intermediate. We performed screening of TAs with high specificity for benzylamine and optimized the reaction parameters such as pH, buffer system, and amine donor concentration. The inhibition of TAs or lipase by benzaldehyde was resolved by the application of an AHR/FDH system, which efficiently improved product formation. The promotor engineering strategy was designed and established for improved control of the expression levels of each biocatalyst, and a single whole-cell system for TA and esterase was developed. Whole-cell biotransformation using various substrate concentrations (10–100 mM **1**) led to the achievement of excellent conversion rates (72–91%) to yield the sitagliptin intermediate. Fine-tuned pH control during the reaction by using a pH-stat system improved the yield by ∼50% at a substrate concentration of 100 mM. The optimized cascade with 100 mM substrate resulted in the achievement of 61% yield, as determined by spectroscopic and chromatographic methods. The protocol developed in this study can be considered an alternative to existing methods, with the advantages of obtainment of a cheap amine donor and simultaneous synthesis of (*R*) and (*S*) enantiomers with the use of a non-chiral amino donor. This novel method can be used to synthesize industrially valuable compounds, such as substituted β- and γ-amino acids, from the corresponding esters as the building blocks of many pharmaceutical compounds.

## Data Availability

The original contributions presented in the study are included in the article/Supplementary Material, further inquiries can be directed to the corresponding author.
