# Supplementary material for: Synthesis of Sitagliptin Intermediate by a Multi-Enzymatic Cascade System Using Lipase and Transaminase With Benzylamine as an Amino Donor
Source: Front Bioeng Biotechnol. 2021 Oct 6;9:757062. doi: 10.3389/fbioe.2021.757062 (PMC8526967; doi:10.3389/fbioe.2021.757062)
Supplement: Supplementary file 1 [file datasheet1.docx]

Supplementary Material

# Supplementary Figures

**Figure S1:** Inhibition of benzaldehyde on the enzymes. Reaction condition: 10 mM pyruvate, 20 mM benzylamine, 0.1 mM PLP, 3 mg_CDW_/mL of TAs, 200 mM Tris-HCl Buffer (pH 7.0), 37℃, 30 min reaction.


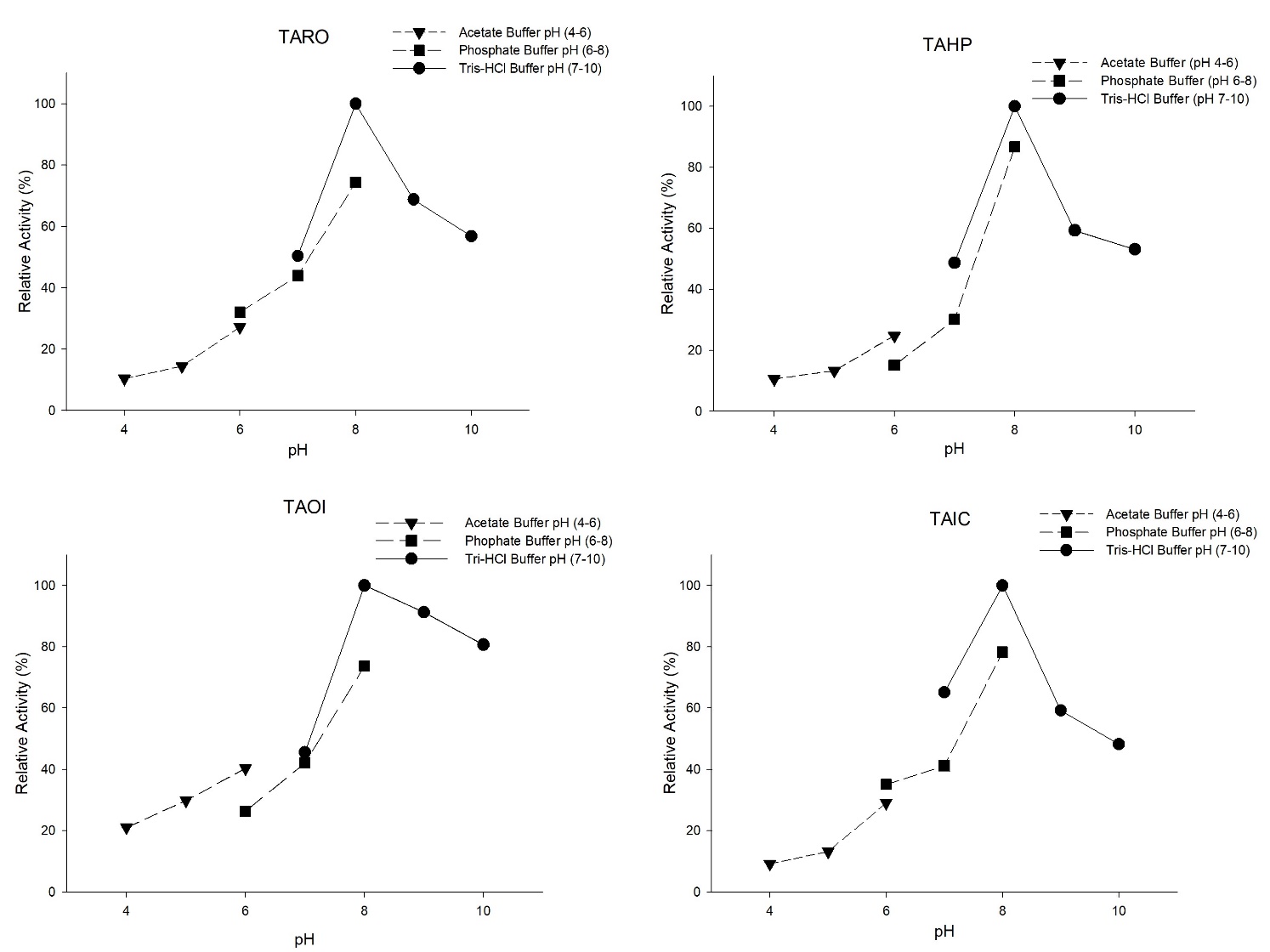


**Figure S2.** Eﬀect of pH on the activity of TARO; TAHP TAOI and TAIC. Reaction condition, 10 mM pyruvate, 20 mM benzylamine, 0.1 mM PLP, TARO; TAHP TAOI and TAIC 0.005 mg/mL, 100 mM Sodium Acetate (pH 4.0-6.0), potassium phosphate (pH 6.0-8.0) and Tris-HCl Buffer (pH 7.0-10.0) and 37℃, 30 min reaction.


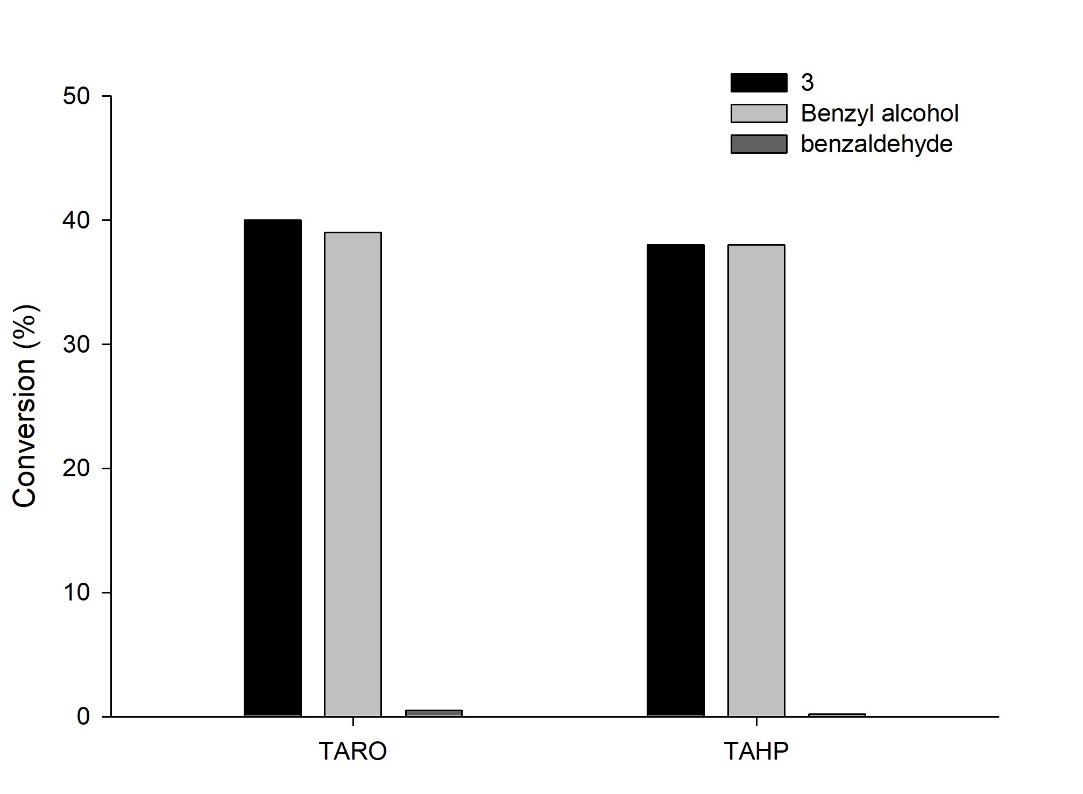


**Figure S3.** Synthesis of sitagliptin intermediate using TA/CRL/AHR/FDH system. Reaction condition:50 mM **1**, 150 mM benzylamine, 40 mg/mL CRL, 27 mg_CDW_/mL TAs, 27 mg_CDW_/mL AHR/FDH cells.


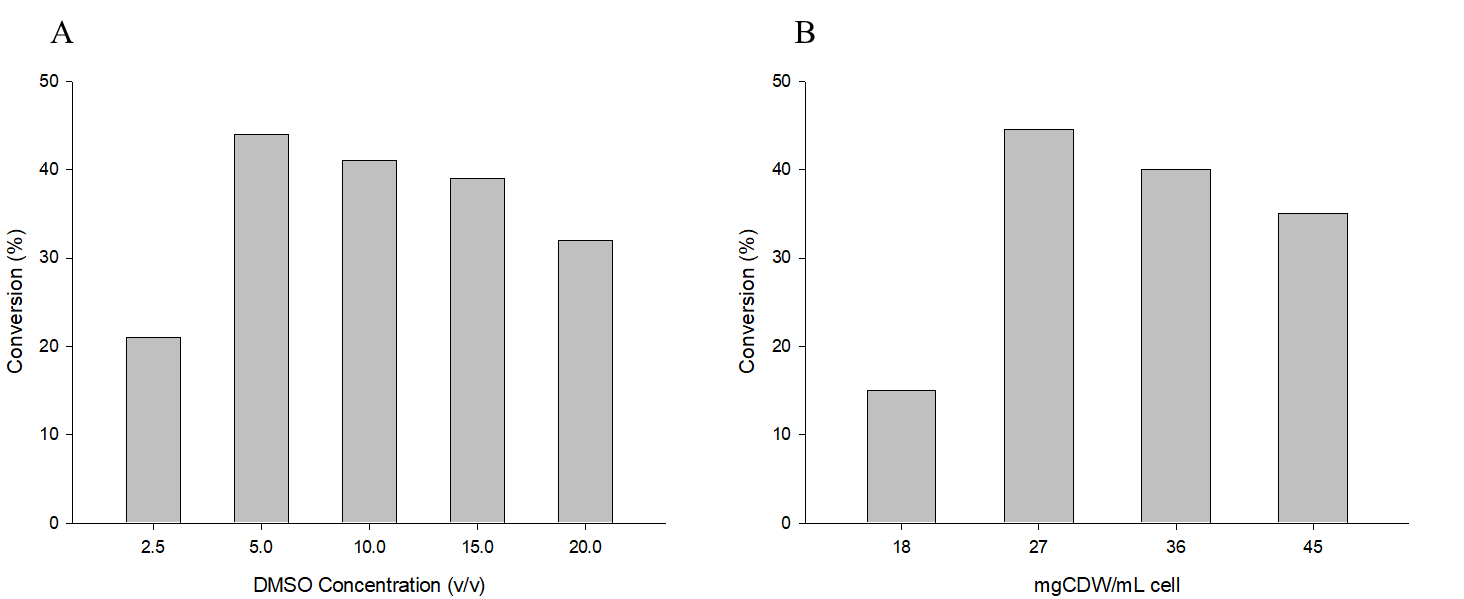


**Figure S4.** A) Effect of DMSO on 50 mM substrate reaction using A4 engineered co-expressed whole cell Reaction condition:50 mM **1**, 150 mM benzylamine, 27 mg_CDW_/mL of TARO-Est PS, 27 mg_CDW_/mL AHR/FDH cells. B) Effect of loading amount of co-expressed whole cell using A4 engineered system. Reaction condition:50 mM **1**, 150 mM benzylamine, 27 mg_CDW_/mL AHR/FDH cells.

**Figure S5.** Time course of 50 mM **1** reaction for synthesis of sitagliptin intermediate. Reaction condition; 50 mM **1**, 150 mM Benzylamine, 0.5 mM PLP, 27 mg_CDW_/mL of TARO-Est, 27 mg_CDW_/mL of AHR & FDH, 100 mM Sodium formate, 200 mM Tris HCl buffer pH 8.0, 37℃, 180 rpm, 24 h.

**Figure S6.** Whole‐cell biotransformation of A1 and A6 engineered systems for the synthesis of **3**. Reaction condition; 50 mM **1**, 150 mM Benzylamine, 0.5 mM PLP, 27 mg_CDW_/mL of TARO-Est, 27 mg_CDW_/mL of AHR & FDH, 100 mM Sodium formate, 200 mM Tris HCl buffer pH 8.0, 37℃, 180 rpm, 24 h.

**Figure S7.** Whole‐cell biotransformation of 300 mM reaction for the synthesis of **3** using A4 engineered system. Reaction condition: 200 mM Substrate, 0.5 mM PLP, 45 or 60 mg_DCW_/mL of TARO-Est PS, 45 or 60 mg_DCW_/mL of AHR & FDH, 200 mM Sodium formate, 200 mM Tris HCl buffer pH 8.0, 37℃, 180 rpm, 24 h.


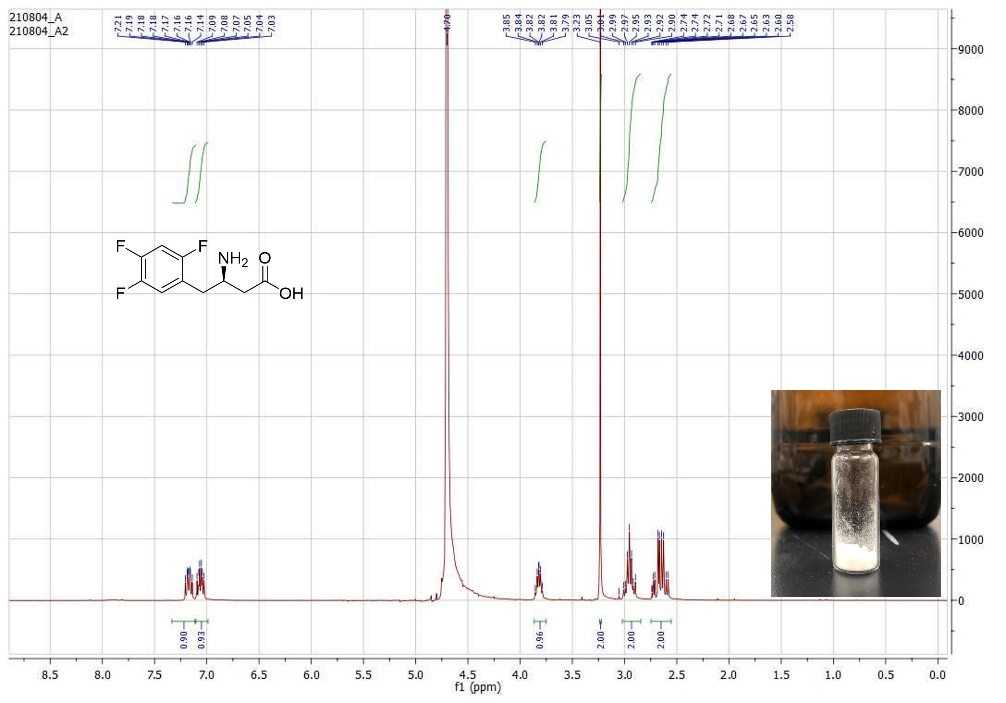


**Figure** **S8**. ^1^H NMR spectrum of Sitagliptin intermediate (**3**).

^1^H NMR (400 MHz, D_2_O δ): 7.14~7.21 (m, 1H), 7.03~7.09 (m, 1H), 3.79~3.85 (m, 1H), 3.23 (s, 2H), 2.92~3.05 (m, 2H), 2.58~2.90 (m, 2H)


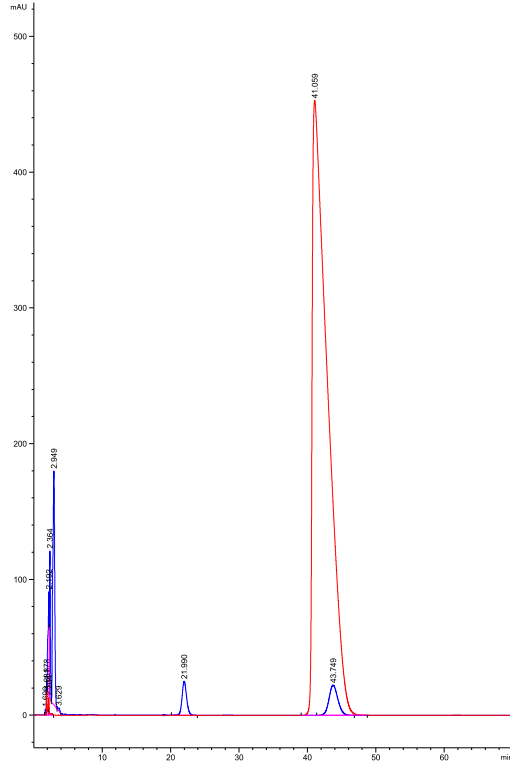


**Figure S9**. HPLC chromatogram for **3** standard (red) and isolated product (blue).


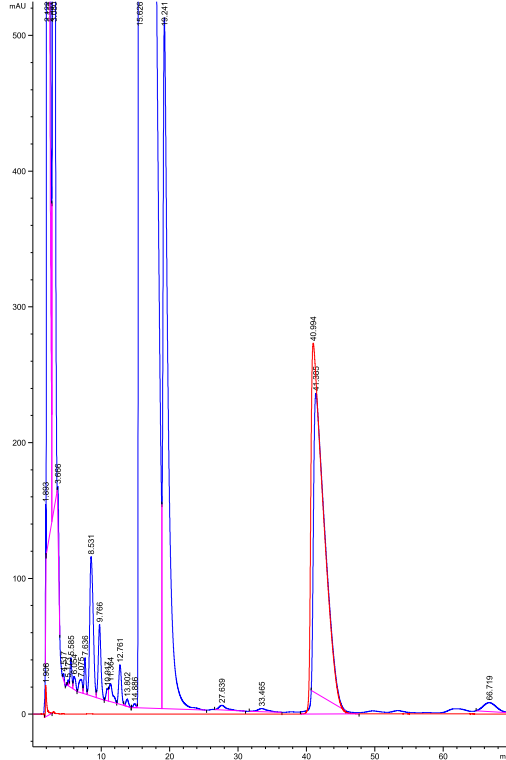


**Figure S10**. HPLC chromatogram for **3** standard (red) and reaction mixture (blue).


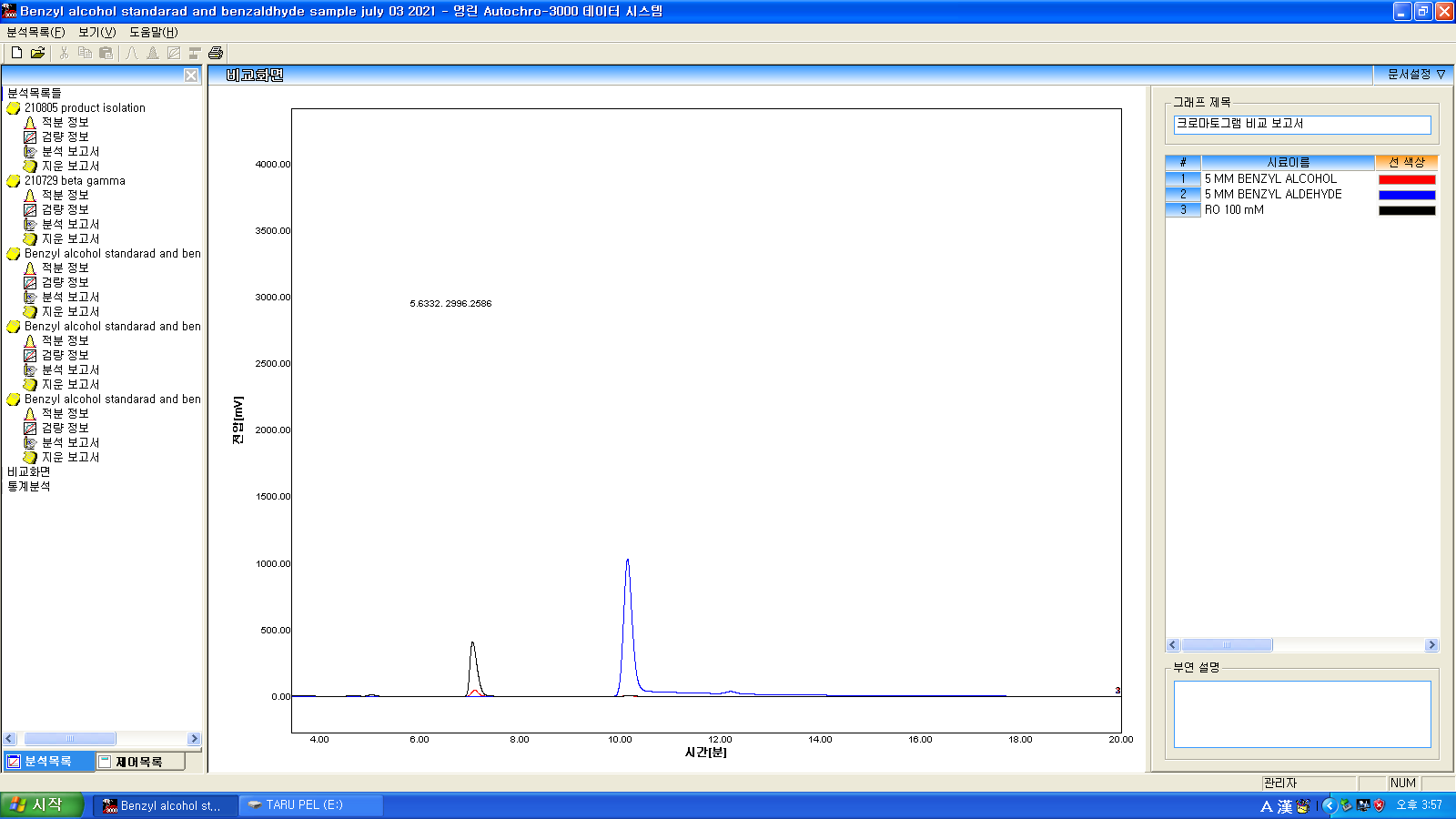
 **Figure S11**. HPLC chromatogram for benzyl alcohol standard (black), benzaldehyde standard (blue)) and reaction mixture (red).

**Table S1**: Effect of amino donor on (*R)*-TA

| Enzyme | Amino donor | Conversion (%) |
| --- | --- | --- |
| TANF | Benzylamine | 18 |
|  | D-alanine | 0.05 |
|  | Isopropyl amine | 0.02 |

Reaction condition: 10 mM acetophenone, (30 or 130 or 100 mM) benzylamine or D-alanine or Isopropyl amine 0.5 mM PLP, 9 mg_DCW_/mL of TANF, 9 mg_DCW_/mL of AHR & FDH or AlaDH & FDH, 50 mM Ammonium or sodium formate, 200 mM Tris HCl buffer pH 8.0, 37℃, 180 rpm, 24 h.
